# Hepatic mitochondrial and peroxisomal alterations in acutely ill malnourished Malawian children: A postmortem cohort study

**DOI:** 10.1016/j.gpeds.2024.100199

**Published:** 2024-09

**Authors:** Catriona M Ling, Tewabu F Sheferaw, Donna M Denno, Dennis Chasweka, Steve B Kamiza, Jaume Ordi, Christopher A Moxon, Kim Kats, Stanley Khoswe, Emmie Mbale, Frank Ziwoya, Abel Tembo, Charalampos Attipa, Isabel Potani, Peter K Kim, James A Berkley, Judd L Walson, Wieger P Voskuijl, Robert H J Bandsma

**Affiliations:** a Department of Nutritional Sciences, University of Toronto, Toronto, Canada; b Translational Medicine, The Hospital for Sick Children, Toronto, Canada; c Amsterdam UMC location University of Amsterdam, Amsterdam Centre for Global Child Health, Emma Children's hospital, Amsterdam University Medical Centres, Amsterdam, the Netherlands; d Department of Pediatrics, University of Washington, Seattle, Washington, USA; e The Childhood Acute Illness & Nutrition (CHAIN) Network, c/o KEMRI Wellcome Trust Research Programme, Nairobi, Kenya; f Department of Global Health, University of Washington, Seattle, Washington, USA; g Department of Paediatrics and Child Health, Kamuzu University of Health Sciences, Blantyre, Malawi; h Department of Pathology, Kumuzu University of Health Sciences, Blantyre, Malawi; i Department of Pathology, Hospital Clinic, Universitat de Barcelona, Spain; j Welcome Centre for Integrative Parasitology, Institute of Infection, Immunity and Inflammation, College of Medical, Veterinary and Life Sciences, University of Glasgow, Glasgow, United Kingdom; k Malawi-Liverpool Wellcome Clinical Research Programme, College of Medicine, University of Malawi, Blantyre, Malawi; l Department of Biomedical Science of Cells and Systems, University of Groningen, University Medical Center Groningen, Groningen, the Netherlands; m Department of Pathology, The Royal (Dick) School of Veterinary Studies and The Roslin Institute, College of Medicine and Veterinary Medicine, University of Edinburgh, Edinburgh, United Kingdom; n Department of Biochemsitry, University of Toronto, Toronto, ON, Canada; o Cell Biology Program, Hospital for Sick Children, Toronto, Ontario, Canada; p Centre for Tropical Medicine & Global Health, Nuffield Department of Medicine, University of Oxford, Oxford, United Kingdom; q Amsterdam UMC location University of Amsterdam, Department of Global Health, Amsterdam Institute for Global Health and Development, Amsterdam University Medical Centres, Amsterdam, the Netherlands

**Keywords:** Severe malnutrition, Mitochondria, Steatosis, Liver, Peroxisomes, Edematous malnutrition, Severe wasting, Kwashiorkor, Marasmus, Minimally invasive tissue sampling

## Abstract

**Objectives:**

To describe and compare liver mitochondrial and peroxisomal histopathology by nutritional status in children who died following hospitalization for acute illness in Malawi.

**Methods:**

Liver tissue was collected using Minimally Invasive Tissue Sampling from eleven children under-five years old who died during hospitalization and were either non-wasted (*n* = 4), severely wasted (*n* = 4) or had edematous malnutrition (*n* = 3). Histology was assessed on hematoxylin and eosin stained slides. Mitochondrial and peroxisomal ultrastructural features were characterized using electron microscopy (EM) and immunofluorescence (IF).

**Results:**

Hepatic steatosis was present in 50 % of non-wasted and severely wasted children and all children with edematous malnutrition. Edematous malnutrition was associated with 56 % and 45 % fewer mitochondria than severe wasting (*p* < 0.001) and no wasting (*p* = 0.006), respectively, and abnormal mitochondrial morphology compared to severe wasting (*p* = 0.002) and no wasting (*p* = 0.035). Peroxisomal abundance was reduced in edematous malnutrition compared to severe wasting (*p* = 0.005), but did not differ from no-wasting.

**Conclusion:**

Edematous malnutrition is associated with reduced abundance and altered morphology of hepatic mitochondria and peroxisomes. Interventions targeting improvements in hepatic metabolic function may be beneficial in improving metabolism and reducing mortality in children with severe malnutrition, particularly in those with nutritional edema.

## Highlights

• Mechanisms leading to death in severe malnutrition are not well understood. This study uses tissue collected through MITS to analyse subcellular defects in hepatic tissue that may be associated with severe malnutrition.
• Abnormalities in hepatic mitochondrial structure and function are present in malnourished children who die in hospital, in particular those with edematous malnutrition, suggesting a possible pathway involved in energy metabolism for future intervention to prevent death in this high-risk population.
• Hepatic mitochondria are altered in children with severe malnutrition who die in hospital, particularly those with edematous malnutrition.

## 1 Introduction

Despite its immense impact on childhood mortality, the number of children suffering from severe malnutrition (SM) has remained relatively stable over the last 5 years.^1^ Inpatient mortality rates among children with SM can reach up to 30 % in sub-Saharan Africa despite adherence to current management guidelines, suggesting improvement of the hospital treatment protocols are needed.^2^, ^3^, ^4^ Malnourished children are more likely to die from concomitant infections such as pneumonia, malaria, and diarrhoea compared to children with better nutritional status.^5^ SM is classified in three phenotypes: severe wasting (defined as MUAC <11.5 cm among 6–59 month olds or height-for-age Z score <−3) (marasmus), the presence of nutritional edema (kwashiorkor), or a combination of both (marasmic-kwashiorkor).

Nutritional edema has been associated with the presence of hepatic steatosis (fatty liver) characterized by altered dynamics in lipid uptake, secretion, and metabolism.^6^, ^7^, ^8^, ^9^, ^10^ Mitochondria and peroxisomes are central regulators of cellular metabolism and cell signalling pathways,^11^, ^12^, ^13^, ^14^ and it has been hypothesized that a lack of functional peroxisomes in the hepatocytes of severely malnourished children may contribute to the development of fatty liver.^15^ Using rodent models of severe malnutrition, we determined that hepatic steatosis is related to impaired mitochondrial function and loss of functional peroxisomes.^16^ In a recent study, our group determined that inpatient mortality in children with severe malnutrition is related to disturbances in cellular energy homeostasis, consistent with impairments in mitochondrial function.^17^ However, a definitive assessment of the state of hepatocyte mitochondria in children with severe malnutrition is lacking. In one historical postmortem study among eight severely malnourished children, the authors identified changes in two hepatic cellular organelles: morphological changes in mitochondria and a reduction in the presence of peroxisomes. This study was largely qualitative. Quantitative analyses on mitochondrial and peroxisomal morphology or abundance was not performed, and the study lacked a comparison group.

The Childhood Acute Illness & Nutrition (CHAIN) Network was established in 2016 to identify biological and socioeconomic risks and pathways to mortality in acutely ill, young children in low- and middle-income countries (LMICs).^18^ At nine sites across six LMICs, children between one week and two years old who were admitted to healthcare facilities due to acute illnesses with or without malnutrition were enrolled. One CHAIN site is Queen Elizabeth Central Hospital (QECH) in Blantyre, Malawi, where a substudy, minimally invasive tissue sampling (MITS), was performed^19^ to improve our understanding of causes of death (CoD) in hospitalized children. MITS involves the use of needles to transcutaneously sample organs and bodily fluids, circumventing the need for incisional autopsy. MITS have been validated compared to incisional autopsy,^20^ found to be more socially acceptable, and is increasingly being deployed in research studies in LMICs.^19^, ^20^, ^21^, ^22^

Since few studies have investigated the association between liver pathology and different forms of severe malnutrition, this study aimed to identify histopathologic mitochondrial and peroxisomal changes postmortem in the livers of children with different forms of severe malnutrition. An improved understanding of pathophysiologic events may allow for the development of novel targeted treatments for highly risk children in these settings.

## 2 Methods

### 2.1 Study design and setting

MiM was a prospective cohort study, hosted at the QECH in Blantyre, Malawi, a national referral and teaching hospital.^18^^,^^22^^,^^23^ This MITS in Malawi (MiM) study initially was focused on recruiting children who were enrolled in CHAIN at QECH who died during hospitalization. However, because of lower than anticipated enrolment and case fatality in this restricted group, the MiM study was expanded to instead include any QECH inpatient deaths in children aged 1 week-59 months with an acute illness (including children enrolled in one of two other QECH-based studies and children in the general pediatric wards).^19^

### 2.2 Participant selection and recruitment

Children aged between 1 week and 59 months old who died during inpatient admission for acute illness and/or SM were eligible for enrolment. Exclusion criteria were: injuries, congenital syndromes, surgical conditions and known terminal illnesses.^19^ Recruitment to MiM was from August 20, 2018 to April 9, 2020. After recruitment the included children were stratified by nutritional status: non-wasted children, children with severe wasting, or children with edematous malnutrition.

Parents/guardians of children eligible for the study were approached by study staff after a respectful period following the child's death. After the parent/guardian provided written informed consent, their child was included in the study. All approached parents/guardians, regardless of an agreement to participate, were assisted with coffin purchase and transportation, and were offered grief support.

### 2.3 Sampling procedures

The MITS procedure was performed as soon as possible after consent was provided and was conducted by an experienced pathologist (SK), at the QECH mortuary per the protocol detailed by Castillo et al.^24^ Briefly, using sterile 14–16 G needles entering at the anterior right axillar line, 11th-12th intercostal space, multiple liver biopsies were collected and placed in either 10 % neutral buffered formalin for fixation prior to paraffin embedding. Sections were stained with heamtoxylin and eosin (H&E). Other biopsies were placed in 2 % glutaraldehyde and 2 % paraformaldehyde in 0.1 M Cacodylate buffer for fixation for electron microscopy and left at 4′C until further processing occurred.

### 2.4 Data collection

Admission clinical data was extracted from medical records (general ward patients) and case report forms from the MiM CHAIN study if co-enrolled.

Determination of nutritional status was based on postmortem anthropemtric measurements and antemortem assessment of edema. Postmortem anthropometry was used since the antemortem data was incomplete in some cases and because postmortem anthropometry is standard in MITS studies.^19^ At the start of the MITS procedure, two study staff members measured mid-upper arm circumference, weight, and length and where there was a discrepancy, measured a third time. The average of two closest measurements was used. *Z* scores were calculated using World Health Organization Anthro software.^25^ SM was defined by mid-upper arm circumference <11.5 cm (among ≥6-month-old children), weight-for-length *z* score <−3 (severe wasting), or nutritional edema, defined as the presence of severe bilateral edema without another known etiology^26^). CoD was determined based on the Child Health and Mortality Prevention Surveillance (CHAMPS) network approach.^23^^,^^27^

### 2.5 Sample selection

**Fig. 1:**
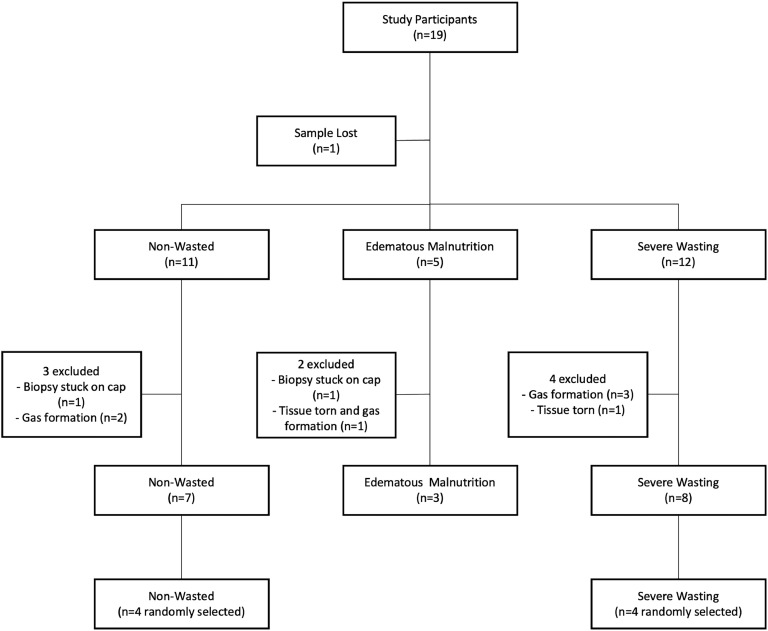
Study flow diagram. Some samples were not included due to complications in processing and fixation for EM: biopsy stuck on the cap of fixation vial during transport (was not immersed in fixation medium), gas formation within tissue during fixation and transport, and tissue biopsy torn and deemed unusable.

Samples that arrived in the laboratory for electron microscopy (*n* = 18) were examined by a laboratory analyst for damage during transport or fixation to determine if the quality was sufficient for electron microscopy imaging, resulting in the exclusion of some cases as shown in Fig. 1. Within each nutritional strata four cases were randomly selected for this current study, with the exception of the edematous malnutrition group where only three samples were available after the prior exclusion.

### 2.6 Histology

H&E slides were assessed by one pathologist (SW) and findings were recorded on standardized case report forms, including for: autolysis, hepatitis, fibrosis, cholestasis, granulomas steatosis, sinusoidal inflammation, and portal inflammation. The latter three features were graded as follows: 0 - none; 1 – mild, focal; 2 – mild, diffuse; 3 – moderate, focal; 4 – moderate, diffuse; 5 – severe, focal; 6 – severe, diffuse.

### 2.7 Immunofluoresence

To visualize the changes in peroxisomal and mitochondrial morphology and abundance, 4 µm paraffin embedded sections were stained by immunofluorescence for Heat Shock Protein 60 (HSP60), an inner mitochondrial membrane protein, and peroxisome membrane protein 70 (PMP70), a peroxisomal membrane protein. Tissue slides were deparaffinized with xylene, rehydrated through an ethanol gradient, and washed in Phosphate-Buffered-Saline (PBS). Antigen retrieval was performed using heat-induced sodium citrate buffer for HSP60 or TRIS-EDTA buffer for PMP70, prior to primary antibody incubation (HSP60 or PMP70) overnight at 4 °C. Following incubation, slides were washed with PBS and subsequently incubated with secondary antibody and DAPI nuclei counterstain for one hour at room temperature in the dark. Slides were mounted with an antifade mounting medium. Imaging was performed on Zeiss-LSM-980 with Airyscan-2 confocal microscope, 63X objective, (Zeiss Inc.). Quantification of area of fluorescence (PMP70 or HSP60) was performed using Fiji software^28^ and was normalized to number of nuclei within the region of interest where five images were analysed in a total of 3 patients per group.

### 2.8 Electron microscopy

Samples were processed and imaged using large-scale electron microscopy (‘Nanotomy’), as described elsewhere.^29^ In brief, in the mortuary samples were fixed in 2 % glutaraldehyde or 2 % paraformaldehyde in 0.1 M Cacodylate buffer (pH 7.4) from the moment the MITS procedure was done between October 9th 2018 and March 2nd 2020 and until the samples were processed for EM between June 10th 2020 and July 7th 2021. The samples were osmicated prior to embedding with 1.5 % osmium tetroxide/potassium ferrocyanide, and ultrathin (80 nm) sections were placed on single slot (2 × 1 mm) copper grids and contrasted with Neodymium.^30^ Images were acquired on a Zeiss Supra55 ATLAS. Complete scanned images of the samples were uploaded online to www.nanotomy.org/OA/Sheferaw2023SUB. For every case, five random sections were selected, each containing 4–15 cells and within each section mitochondrial abundance, shape, and size was measured by a research student using Fiji, an opensource image processing package in ImageJ.^28^ Mitochondrial abundance was determined by counting the number of mitochondria and normalizing to size selection area, mitochondrial shape was quantified by measuring the circularity index (*circularity= 4pi(area/perimeter^2^),* and mitochondrial size was quantified by measuring the area of each individual mitochondrion.

### 2.9 Statistical analysis

Means, median (range), percent/number of cases, and IQR were calculated in Microsoft Excel. All other statistical analyses were performed using GraphPad Prism 9.3.1. Between group comparisons were performed by one-way analysis of variance (ANOVA) with Tukey post hoc test, as the data was normally distributed. Statistical significance was defined by 2-tailed *p* < 0.05.

### 2.10 Ethical considerations

The Malawi National Health Sciences Research Committee (NHSRC 1913), the Hospital for Sick Children Research Ethics Board (1,000,064,008), and Oxford Tropical Research Ethics Committee, UK (OxTREC 34–16) provided eithical approval. The University of Washington Institutional Review Board exempted the study from review (STUDY00003689).

## 3 Results

### 3.1 Patient characteristics

Eleven children were included, two with edematous malnutrition, four with severe wasting and four non-wasted. One child (case #024) with mixed SM (edematous and severe wasting) was analayzed in the edematous malnutrition group. Mean age at death was 15 months, with a range of 1 week to 59 months (Table 1). Length of hospital stay until death varied from 0 to 18 days (median of two days) and the median postmortem interval (time between death and the MITS procedure) was 7 h (range 2–19). Three of the four children with severe wasting were HIV infected (positive rapid-antibody test), with two already on antiretroviral therapy (ART). Among the non-wasted children, one tested HIV-positive. One child with edematous malnutrition was on ART and had a positive rapid antibody-based test. Malaria testing was done postmortem, one child with edematous malnutrition (case 013) tested positive for malaria. The most prevalent immediate causes of death were pneumonia (27 %), gastroenteritis (27 %) and sepsis (18 %).

**Table 1:** Characteristics of Study Participants & Pathologic Hepatic Features (*n* = 11).

			Characteristics of Study Participants								Pathologic Hepatic Features			
Nutritional Status^a^	Case Number	Age, mo	Sex	HIV infected	PM anthropometric measurements b			LOS, days	PMI, hrs	Immediate COD	Steatosis		Inflammation	
					MUAC (cm)	WHZ	HAZ				Present	Degree	Present	Degree
Non-wasted	025	2	F	No	11.2	−0.53	−1.19	8	19	Pneumonia due to Klebsiella pneumoniae	no	–	no	–
	028	7	F	Yes	13.6	0.50	−1.28	0	6	Pulmonary haemorrhage	yes	4	yes	4
	009	0.25	M	No	9.7	−0.49	−2.50	2	2	Disseminated intravascular coagulation	no	–	no	–
	022	17	F	No	14.0	−1.01	−0.27	0	17	Gastroenteritis	yes	4	yes	4
Edematous Malnutrition	013	24	F	No	12.6	−2.94	−1.29	1	7	Pneumonia due to Haemophilus influenzae, Streptococcus pneumoniae	yes	6	yes	6
	023	59	F	No	11.3	−1.91	−5.58	9	4	Pneumonia due to Streptococcus pneumoniae, Haemophilus influenzae, Klebsiella pneumoniae	yes	4	yes	4
	024	5	F	No d	6.7	−5.61^c^	−5.64	1	2	Sepsis due to Klebsiella pneumoniae	yes	1	yes	1
Severe Wasting	002	12	M	Yes e	10.0	−4.05	−4.15	18	4	Gastroenteritis	yes	6	yes	6
	003	15	F	Yes e	10.8	−3.69	−2.58	2	10	Gastroenteritis	yes	5	yes	5
	006	22	M	No	10.6	−3.74	−3.05	8	11	Sepsis due to Escherichia coli	no	–	no	–
	007	4	M	Yes f	9.9	−4.70	−2.00	13	9	HIV disease resulting in haematological abnormalities, NOS	yes	6	yes	6

Abbreviations: HIV, human immunodeficiency virus; NR, non-reactive; R, reactive; PM, postmortem; MUAC, mid-upper arm circumference (cm); WHZ, weight-for-height z-score; LOS, length of hospital stay in days (time difference between ante-mortem and postmortem measurement); PMI, postmortem interval in hours (time from death to initiation of minimally invasive tissue sampling procedure); COD, cause of death; MUAC, mid-upper arm circumference; WHZ, weight-for-height z-score; HAZ, Height for Age Z-score; LOS, length of hospital stay in days (time difference between antemortem and postmortem measurement); PMI, postmortem interval in hours (time from death to initiation of minimally invasive tissue sampling procedure); NOS, not otherwise specified. Descriptive findings from H&E stained livier sections. No sections were found to have autolysis. The presence, degree, and severity of steatosis was determined, as was the presence and location of inflammation, fibrosis, and cholestasis. Steatosis severity score: 0 - none; 1 – mild, focal; 2 – mild, diffuse; 3 – moderate, focal; 4 – moderate, diffuse; 5 – severe, focal; 6 – severe, diffuse. No autolysis, hepatitis, or granulomas were found in any samples.

a Based on postmortem anthropometric measurements and antemortem assessment of edema.

b Postmortem World Health Organisation defined anthropometric status. Based on postmortem anthropometric measurements.

c This patient has mixed SM but was analyzed in the edematous malnutrition group.

d Reactive, but unclear from clinical notes if child is actually HIV positive or on ARTs for prevention of mother-to-child transmission (PMTCT) of HIV. PCR negative per postmortem data.

e On Antiretroviral Therapy (ART).

f Newly diagnosed HIV positive on admission.

### 3.2 Hepatic steatosis is observed in children with severe malnutrition

**Fig. 2:**
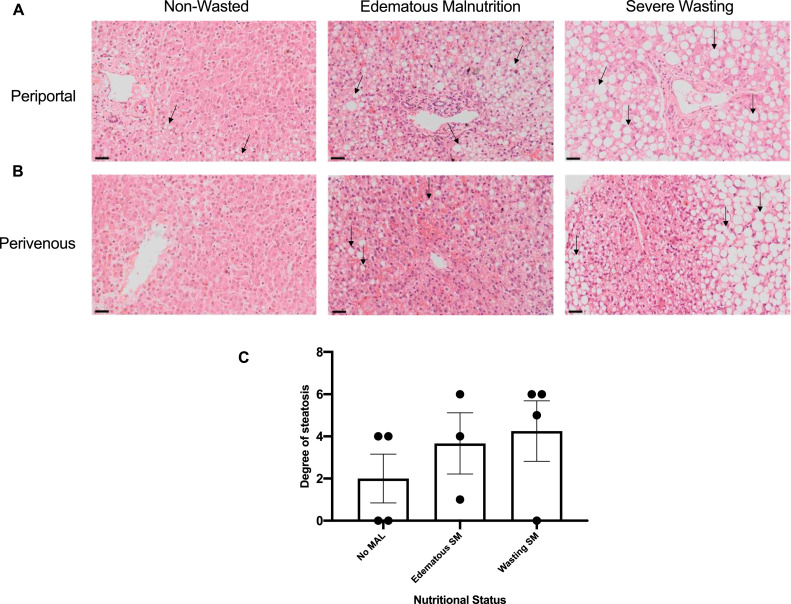
Hepatic Histology. (A) Representative H&E images of the hepatic periportal region from cases 022 (non-wasted), 023 (Edematous SM), 003 (Severe wasting). Scale = 92.5um (B) Representative H&E images of the hepatic perivenous region from cases 022 (non-wasted), 023 (Edematous SM), 002 (Severe wasting). Black arrows point to examples of steatosis. Scale = 92.5um (C) Degree of steatosis plotted per patient for each nutritional status.

Hepatic steatosis was observed in all three cases of edematous malnutrition and in three of four children with severe wasting and two in four without wasting. The degree of steatosis varied both across and within the three nutritional strata and are summarized in (Table 1, Fig. 2a,b).

### 3.3 Hepatic mitochondrial abundance and morphology are affected by severe malnutrition

**Fig. 3:**
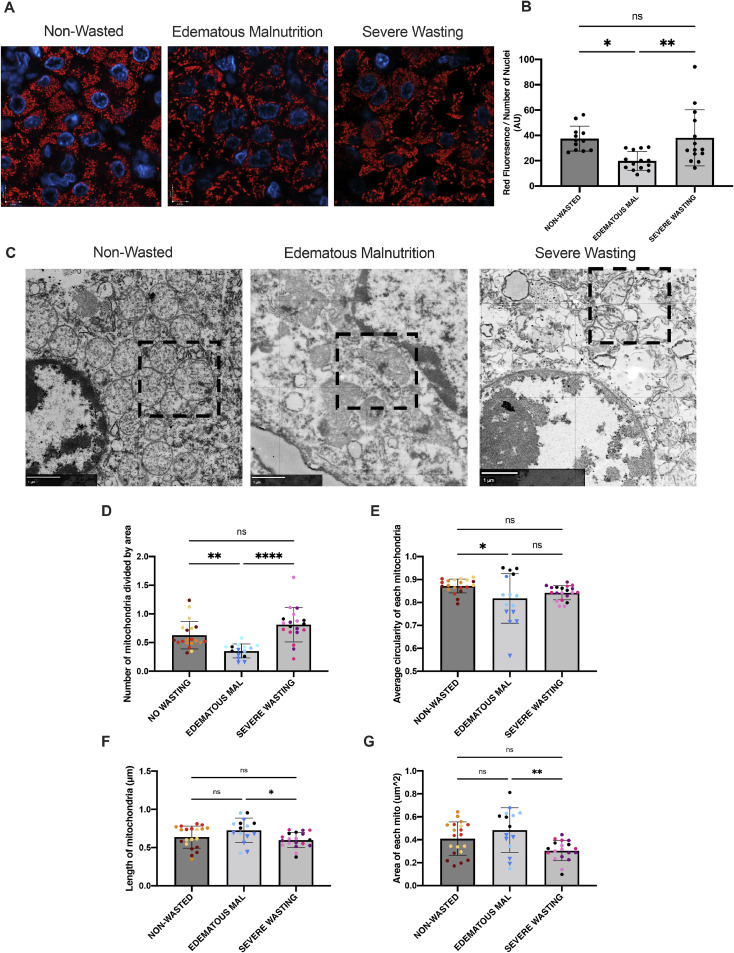
Mitochondrial morphology and abundance are altered in children with SM. (A) Representative immunofluorescent images of HSP60 (red) and DAPI (blue). Scale = 6.2um. (B) Quantification of immunofluorescent images where area of red fluorescence (AU) was normalized to number of nuclei (*n* = 5 images in *N* = 3/group). (C) Representative images of mitochondria within hepatocytes visualized through electron microscopy. Inset highlights mitochondria. Scale = 1um. (D-G) corresponding quantification of mitochondria within the electron microscopy image where (D) is the number of mitochondria normalized to area of the cell quantified, (E) is the average circularity index of mitochondria within a hepatocyte, (F) is the average length of a mitochondria within a hepatocyte, and (G) is the average area of a mitochondria within a hepatocyte. Dot colours represent different cells from the same patient, where 4–15 cells, identified by nuceli, were analyzed in a total of 3–4 patients per group. Triangular points in the in the edematous malnutrition group correspond to the patient with mixed severe malnutrition.

Staining for Heat Shock Protein 60 (HSP60), an inner mitochondrial membrane protein, showed a significant reduction in area of fluorescence in the edematous malnutrition group compared to the non-wasted group (*p* = 0.01) (Fig. 3a & 3b). To corroborate this, we determined the number of mitochondria normalized to cell area, which was reduced in the children with edematous malnutrition compared to those without wasting (*p* = 0.005) and with severe wasting (*p* = 0 < 0.0001) (Fig. 3c & 3d). Mitochondrial abundance was higher in the wasted group compared to the non-wasted group through electron microscopy (*p* = 0.056), however no change in abundance was observed through HSP60 immunofluorescence.

Next, we assessed mitochondrial morphology. EM analysis highlighted that many of the mitochondria in children with edematous malnutrition appeared to have an abnormal shape compared to the homogenous, circular population of mitochondria in the group with non-wasted children (Fig. 3c). This observation was confirmed using quantification of the degree of circularity of each individual mitochondrion (Fig. 3e). Mitochondria from the edematous phenotype deviated from a circular shape to a larger extent compared to the non-wasted group (*p* = 0.03). The severe wasting group had mitochondria that were not significantly different in circularity compared to the other groups. In addition to the level of circularity of the mitochondria, the area and length of each individual mitochondrion was quantified to further interpret their morphology (Fig. 3f,g). The area of the mitochondria in the edematous group was significantly larger compared to the wasted group (*p* = 0.002). The length of the mitochondria was not altered in the children with edematous malnutrition compared to the no-wasting group but mitochondria were significantly longer in comparison to the severe wasting group (*p* = 0.023). The severe wasting group showed no significant alterations in area or length of mitochondria in comparison to the no-wasting group.

### 3.4 Hepatic peroxisomal abundance is reduced in edematous malnutrition

**Fig. 4:**
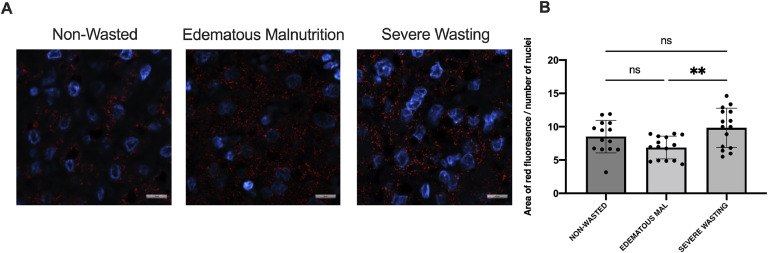
Peroxisomal abundance is altered in children with edematous malnutrition. (A) Representative immunofluorescent images of PMP70 (red) and DAPI (blue). Scale = 1um. (B) Quantification of immunofluorescent images where the area of red fluorescence (AU) was normalized to number of nuclei (*n* = 3/group).

Since peroxisomes and mitochondria are tightly linked organelles and peroxisomes have been reported to be affected by SM in pre-clinical models,^16^^,^^31^^,^^32^ we next visualized the peroxisomes and determined changes in abundance. Overall, hepatic peroxisomes, visualized through peroxisome membrane protein 70 (PMP70), appeared to be reduced in abundance in patients with edematous malnutrition compared to those with severe wasting (*p* = 0.005), however no differences were seen compared to the patients without wasting (Fig. 4).

## 4 Discussion

In this postmortem study of children with different nutritional states, we report three important findings. First, we have shown that steatosis can be found in both forms of severe malnutrition as well as non-wasted children. This is in contrast to prior literature attributing hepatic steatosis primarily to the edematous phenotype of severe malnutrition.^9^^,^^10^^,^^33^ Second, we show that mitochondria have a lower abundance and are larger in children with edematous malnutrition. Lastly, lower numbers of peroxisomes were seen in the edematous malnutrition group. Our study underscores that severe malnutrition is associated with distinct disruptions in cellular organelle biology in the liver. These findings may ultimately have important implications for interventions to address causes of mortality in these vulnerable children. Despite the prevalence of severe malnutrition and its strong association with mortality in children under five years of age, there is still limited understanding of the specific cellular and metabolic dysfunctions, accompanied by a lack of targeted interventions.^34^ This is especially crucial with respect to liver function, as this organ is essential for metabolism of nutrients and energy homeostasis.^35^^,^^36^

This study presents an analysis of hepatic mitochondria and peroxisomes in children who died from an acute illness with severe wasting and no wasting using H&E, electron microscopy and immunofluoresence. To our knowledge, this is the first paediatric use of minimally invasive liver tissue sampling, electron microscopy, and immunofluorescence staining of mitochondria of the liver in children dying of an acute illness with edematous malnutrition, severe wasting and no wasting. A study published in 1933 describing the postmortem findings in three patients suffering from edematous severe malnutrition identified the presence of a fatty liver or hepatic steatosis, which has also been described in subsequent studies published in 1948 and 1964 and has since become a defining organ level disturbance in edematous malnutrition (kwashiorkor).^9^^,^^10^^,^^33^ While the presence of hepatic steatosis has more frequently been described in children with edematous malnutrition,^9^^,^^10^^,^^33^ it has also been noted to be present to a lesser degree in children with severe wasting.^37^ We found hepatic steatosis in patients with severe wasting and in two children without wasting. However, as hepatic steatosis is also a feature observed in sepsis, we cannot definitively attribute the cause to malnutrition.^38^^,^^39^ Furthermore, all patients with severe wasting that had hepatic steatosis were concomitantly infected with HIV, as was the non-wasted patient with hepatic steatosis. HIV has been reported to contribute to the development of hepatic steatosis in association with multiple risk factors, including the risk factors for metabolic syndrome (dyslipidemia, increase waist circumference, and insulin resistance), HIV-related lipodystrophy, genetic polymorphisms, antiretroviral medications and the gut microbiome.^40^^,^^41^ While these factors are more likely to play a role in hepatic steatosis in long-term HIV-infected patients who are older than those in the present study,^40^^,^^41^ which are less than two years of age, we cannot exlude the possibility that HIV could have played a contributing role in the steatosis found in the HIV positive patients. Literature is limited, however, on hepatic subcellular defects among severely malnourished patients. One study by Brooks et al. 1994^42^ in eight Jamaican children with edematous malnutrition assessed liver tissue by electron microscopy and documented various ultrastructural abnormalities, including reduced peroxisomes and swollen mitochondria. Our study confirms these findings. Enlarged mitochondria may be indicative of a hyperfused state, which has previously been described as a mechanism to protect them from degredation under conditions of starvation and in doing so preserve energy production capacity of the cell for as long as possible.^43^ We further elaborate on these previous findings by describing reduced mitochondrial abundance in children with edematous severe malnutrition both through EM analysis and HSP60 immunofluoresence, as the area of mitochondrial protein staining correlates with the overall mitochondrial mass. We also observed that peroxisomes, an organelle known to be strongly linked to the mitochondria, were reduced in the livers of children with edematous malnutrition. We also observed that patients with severe wasting showed a potential trend towards a numerically higher average mitochondrial and peroxisomal abundance compared to children without severe wasting. We hypothesize that this potential increase in organelle abundance in patients with severe wasting is related to a dysfunction in the pathway that clears damaged and dysfunction organelles from the cell, known as autophagy. Supporting this hypothesis is the observed numerical reduction in the size of the mitochondria in the severe wasting group compared to the non-malnourished children. When mitochondrial are designated for autophagic degradation, they will undergo a process known as fission, wherein the mitochondria will fragment to facilitate their degradation. Autophagic dysfunction and consequent changes in organelle abdunance and morphology in response to severe malnutrition have been described in numerous preclinical studies,^16^^,^^31^^,^^32^ further supporting the aforementioned hypothesis.

A significant obstacle to study hepatic cellular and metabolic pathology in children with severe malnutrition has been the ability to acquire human tissue samples. Therefore, this has been mainly studied in pre-clinical SM models. A study by our group found that rodents fed a protein-restricted diet had hepatic steatosis and reduced mitochondrial abundances, more deformed mitochondria, and reduced mitochondrial function compared to those fed a control diet.^32^ While we could not directly measure mitochondrial function in the current study, it is well-known that abnormal mitochondrial structure can be associated with suboptimal mitochondrial function.^44^ For example, disruption of the inner mitochondrial membrane alters the mitochondrial membrane potential and greatly reduces the functional capacity of the electron transport chain.^45^ Furthermore, modulation of mitochondrial surface area disrupts the balance between the arrangement for optimal energy conversion and surface area for interaction with other organelles.^43^ Finally, larger mitochondria require more energy to degrade, resulting in their preservation and maintenance within the cell. While this is an adaptive response to acute starvation, under conditions of chronic starvation this may become maladaptive as damaged mitochondria may not be removed and regenerated.

The results in our study are consistent with recent work where we demonstrated that mortality in children with SM is related to metabolic disturbances indicating mitochondrial dysfunction.^17^ The circulating metabolic profiles of 184 Kenyan and Malawian children with SM differed significantly in children who died compared to those who survived. Amongst these differences was a significant increase in mitochondria-related bioenergetic pathway substrates in children who died, suggesting disturbances in mitochondrial function.^17^ The findings of altered hepatic cell organelles in children dying from an acute illness with forms of SM should direct us towards new therapies aiming at hepatic dysfunction and focusing treatment on restoring mitochondrial health. Recent work in a preclinical model of SM indicated that rescuing mitochondrial function was associated with improvement in the hepatic phenotype, including a resolution of hepatic steatosis.^32^

Our study has several limitations. Our small sample size was limited to 11 children, as we selected a random subset of the MiM patients to include in this analysis hence, our assessment of correlations with liver histology is exploratory. A second limitation to this study is the possibility of confounding factors affecting the liver histology, such as CoD, which has hindered our ability to draw conclusions as to what was caused specifically by malnutrition. Finally, postmortem studies have intrinsic limitations, including the possibility of postmortem alterations in subcellular structures. To address this, we included a group of children without SM. Furthermore, we strived to minimize the postmortem interval before the MITS procedure commenced. Results from a recent study indicate that sufficient quality liver specimens may be retrieved using MITS procedures up to four days after death.^46^ Our postmortem interval ranged from two to nineteen hours, well within the aforementioned limit of four days, supporting our use of these samples for our analysis.

In conclusion, this study is unique in using tissue sampling in children with two forms of SM and children without SM to understand the complexities of hepatic subcellular structures in SM. The findings in this study suggest that interventions targeting altered mitochondrial function in children with SM may be beneficial in reducing mortality in this high-risk population.

## Availability of data and materials

The datasets used and/or analysed are available from the corresponding authors on request.

## CRediT authorship contribution statement

**Catriona M Ling:** Writing – review & editing, Writing – original draft, Visualization, Validation, Project administration, Methodology, Investigation, Funding acquisition, Formal analysis, Data curation, Conceptualization. **Tewabu F Sheferaw:** Writing – review & editing, Writing – original draft, Investigation, Formal analysis, Data curation, Conceptualization. **Donna M Denno:** Writing – review & editing, Validation, Methodology, Investigation, Conceptualization. **Dennis Chasweka:** Writing – review & editing, Methodology, Investigation. **Steve B Kamiza:** Writing – review & editing, Investigation, Data curation. **Jaume Ordi:** Writing – review & editing, Investigation, Formal analysis, Data curation, Conceptualization. **Christopher A Moxon:** Writing – review & editing, Resources, Methodology, Conceptualization. **Kim Kats:** Writing – review & editing, Validation, Resources, Methodology, Investigation. **Stanley Khoswe:** Writing – review & editing, Methodology, Investigation, Data curation. **Emmie Mbale:** Writing – review & editing, Project administration, Investigation, Data curation. **Frank Ziwoya:** Writing – review & editing, Methodology, Investigation. **Abel Tembo:** Writing – review & editing, Methodology, Investigation. **Charalampos Attipa:** Writing – review & editing, Validation, Methodology, Investigation. **Isabel Potani:** Writing – review & editing, Methodology, Investigation. **Peter K Kim:** Writing – review & editing, Supervision, Methodology, Investigation. **James A Berkley:** Writing – review & editing, Supervision, Project administration, Investigation, Funding acquisition, Conceptualization. **Judd L Walson:** Writing – review & editing, Supervision, Methodology, Investigation. **Wieger P Voskuijl:** Writing – review & editing, Validation, Supervision, Resources, Project administration, Methodology, Investigation, Funding acquisition, Conceptualization. **Robert H J Bandsma:** Writing – review & editing, Validation, Supervision, Resources, Project administration, Methodology, Investigation, Funding acquisition, Conceptualization.

## Declaration of competing interest

The authors declare that they have no known competing financial interests or personal relationships that could have appeared to influence the work reported in this paper.
